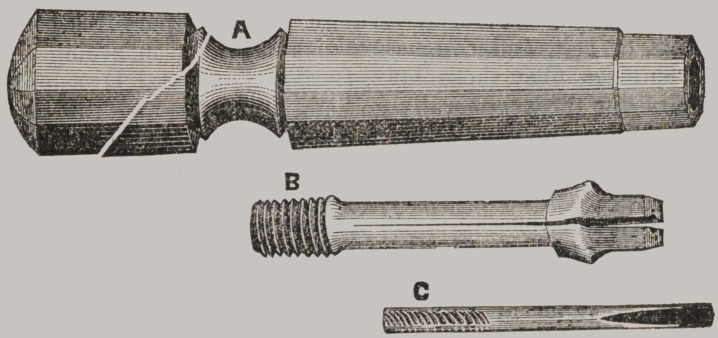# Forbes’ Socket Handle

**Published:** 1858-11

**Authors:** 


### A SOCKET HANDLE.

Below we give a cut of a socket handle used by, and brought to the notice of the profession, by Dr. I. Forbes, of St. Louis. The principle is an excellent one, and should have been employed long ago.

It consists of a screw socket, fixed in a suitable handle; into this socket is screwed that portion of the instrument that grasps the point or cutting portion of the instrument. This has two jaws, vice-like in appearance, with a longitudinal groove in each, so that when the jaws are closed, a square hole is formed, for the reception of the small instruments. The jaws are closed firmly together, or upon any thing placed between them, by being screwed into the socket, it being somewhat beveled, so that as it passes in, it is pressed firmly together.

Dr. Forbes uses many different forms of instruments—cutting instruments in particular. He values very highly, cutting instruments of a gouge form, for opening up cavities, and indeed for forming them.

These socket handles are not made by any dental instrument maker, that we are aware of. They should be made, and for sale by our dental depots. The cut represents all parts of the instrument.

*A* is the handle, which contains the socket. *B* is the portion that screws into the socket, and grasps the small instrument. *C* is the small instrument used with it. T.

Toland furnishes them to the profession.—*Dental Reg.*

## Figures and Tables

**Figure f1:**